# Coadministration of Ketamine and Perampanel Improves Behavioral Function and Reduces Inflammation in Acute Traumatic Brain Injury Mouse Model

**DOI:** 10.1155/2020/3193725

**Published:** 2020-12-10

**Authors:** Faleh Alqahtani, Mohammed A. Assiri, Mohamed Mohany, Imran Imran, Sana Javaid, Muhammad Fawad Rasool, Waleed Shakeel, Farzane Sivandzade, Ahmed Z. Alanazi, Salim S. Al-Rejaie, Musaad A. Alshammari, Fawaz Alasmari, Mohammed Mufadhe Alanazi, Faisal F. Alamri

**Affiliations:** ^1^Department of Pharmacology and Toxicology, College of Pharmacy, King Saud University, Riyadh 11451, Saudi Arabia; ^2^Department of Pharmacology, Faculty of Pharmacy, Bahauddin Zakariya University, Multan 60800, Pakistan; ^3^Department of Pharmacy, The Women University, Multan 60000, Pakistan; ^4^Department of Pharmacy Practice, Faculty of Pharmacy, Bahauddin Zakariya University, Multan 60800, Pakistan; ^5^Department of Pharmaceutical Sciences, Texas Tech University Health Sciences Center, Amarillo, TX 79106, USA; ^6^College of Sciences and Health Profession, King Saud bin Abdulaziz University for Health Sciences, Jeddah, Saudi Arabia; ^7^King Abdullah International Medical Research Center, Jeddah, Saudi Arabia

## Abstract

Traumatic brain injury (TBI) is among the most debilitating neurological disorders with inadequate therapeutic options. It affects all age groups globally leading to post-TBI behavioral challenges and life-long disabilities requiring interventions for these health issues. In the current study, C57BL/6J mice were induced with TBI through the weight-drop method, and outcomes of acutely administered ketamine alone and in combination with perampanel were observed. The impact of test drugs was evaluated for post-TBI behavioral changes by employing the open field test (OFT), Y-maze test, and novel object recognition test (NOR). After that, isolated plasma and brain homogenates were analyzed for inflammatory modulators, i.e., NF-*κ*B and iNOS, through ELISA. Moreover, metabolomic studies were carried out to further authenticate the TBI rescuing potential of drugs. The animals treated with ketamine-perampanel combination demonstrated improved exploratory behavior in OFT (*P* < 0.05), while ketamine alone as well as in combination yielded anxiolytic effect (*P* < 0.05‐0.001) in posttraumatic mice. Similarly, the % spontaneous alternation and % discrimination index were increased after the administration of ketamine alone (*P* < 0.05) and ketamine-perampanel combination (*P* < 0.01‐0.001) in the Y-maze test and NOR test, respectively. ELISA demonstrated the reduced central and peripheral expression of NF-*κ*B (*P* < 0.05) and iNOS (*P* < 0.01‐0.0001) after ketamine-perampanel polypharmacy. The TBI-imparted alteration in plasma metabolites was restored by drug combination as evidenced by metabolomic studies. The outcomes were fruitful with ketamine, but the combination therapy proved more significant in improving all studied parameters. The benefits of this new investigated polypharmacy might be due to their antiglutamatergic, antioxidant, and neuroprotective capacity.

## 1. Introduction

Traumatic brain injury (TBI) is one of the most common reasons behind neurological impairment that continues to distress the masses of the population worldwide. The Centre for Disease Control (CDC) reports that the rate of hospital visits and deaths due to TBI has been continuously increasing with time [1]. The affectees belong to different age groups including children (<5 y), adolescents (16-25 y), and elderly (>60 y) due to accidental collision with some object during falls or traffic-related misfortunes [2]. It can be considered as the “silent epidemic” due to its contribution towards morbidity and mortality worldwide [3]. The brain insult in TBI patients is extensively associated with life-long disabilities as inadequate therapeutic options cannot deal with the array of consequences. Unfortunately, the rehabilitation of such disabled patients places a financial burden on affected families as well as on overall healthcare [4].

Several studies through animal models have demonstrated the deleterious impact of TBI on quality of life due to deteriorated reasoning and learning capabilities [5]. The pathophysiology of TBI can be divided into primary and secondary phases [6]. The primary phase comprises the direct mechanical injury to the brain that damages the cellular membranes, subcellular structures, and blood vessels thus altering cerebral blood flow due to hemorrhage and increased cell death due to ischemia [7]. These events lead to the activation of immune cells residing in the brain, i.e., microglia and astrocytes causing further initiation of inflammation through cytokines and chemotaxis [8].

Nitric oxide (NO) is an inflammatory product that takes part in secondary TBI [9] as well as in the recovery phase [10]. Its detrimental or beneficial role is decided by the site [11], quantity [12], and time [13] of its production. It is synthesized by three isoforms of NOS among which a Ca^+2^-independent type, inducible nitric oxide synthase (iNOS), is not expressed in the brain under normal circumstances. However, any kind of stimuli, i.e., inflammation, injury, or hypoxia, causes the activation of iNOS in neurons and glial cells. In response to certain proinflammatory cytokines, overexpression of iNOS is seen in vascular smooth muscle cells and stimulated macrophages [14] resulting in increased production of NO which causes increased leukocyte accumulation in the brain from 4 h to 7 days of TBI [15]. Gene expression of iNOS requires activation of the transcriptional factor NF-*κ*B [16] which plays a role in inflammation and immunity in the CNS. The previously reported studies demonstrate that inhibition of NF-*κ*B signaling cause reduced inflammation and enhanced neuroprotection in animal models of TBI [17, 18]. Besides these, stimulated microglia cause damage to oligodendrocytes and neurons via expression of iNOS [19]. Various clinical and experimental studies have observed elevated NO levels and overexpression of iNOS in TBI [20]. Furthermore, inhibition of iNOS also resulted in significantly improved clinical outcomes thus suggesting the neuroprotective role of iNOS inhibition after TBI [21].

Another offender of TBI is glutamate, a principal excitatory neurotransmitter in the brain, which [22] plays a crucial role in the pathophysiology of brain trauma during the second phase [23]. Studies have revealed that the raised glutamate levels in humans facing TBI may persist for days or weeks in certain parts of the brain [24]. Increased levels of glutamate after brain trauma cause excitotoxicity resulting in neuronal damage and death and if not regulated can lead to long-term cognitive decline and motor dysfunction [25]. Mostly, the excitatory neurotransmission in the brain is facilitated by AMPA and NMDA receptors and their altered expression is seen in pathological signaling happening during various disease states including TBI [26]. Hence, targeting these receptors can be a therapeutic option for management of brain trauma. Perampanel, marketed as Fycompa, is a noncompetitive antagonist of AMPA receptor [27] and has demonstrated the neuroprotective role in controlled cortical impact model of TBI due to anti-inflammatory and antioxidant potential [26]. Besides this, antagonism of AMPA receptor has been reported to improve cognition and behaviors in rats with TBI [28].

NMDA receptors have been targeted by numerous anesthetic agents, including ketamine. Antagonism of NMDA receptor by ketamine has also been postulated to exert neuroprotection in TBI [29]. Ketamine is also reported to inhibit NO synthesis thus exerting vasoconstrictive impact resulting in hemodynamic stability [30]. It has also shown to affect hippocampal cell proliferation after TBI leading to neurogenesis which led to better learning and behavior in mice with TBI [31].

In this recent study, we examined the therapeutic outcomes of coadministration of perampanel and ketamine in the management of TBI as it has never been addressed previously. Some behavioral experiments were also employed to examine the role of polypharmacy on the cognitive deficit in the mouse model of weight-drop TBI, whereas their impact on local and systemic inflammation was estimated through their effect on iNOS and NF-*κ*B levels. The scientific findings were further evaluated through metabolomics of the animal plasma. We hypothesized the beneficial outcomes by combination therapy of perampanel-ketamine on local and systemic inflammation as well as on behavioral adversity seen in TBI.

## 2. Materials and Methods

### 2.1. Animal

Healthy male C57BL/6J mice of 8-10 weeks (24-28 g) were utilized in this study. The animals were housed in standardized maintenance conditions, i.e., 12 h L/D cycle, 25°C temperature, and provided with free excess of water and a standard mouse chow. The whole animal study was carried out after ethical permission granted by the Research Ethics Committee (REC), King Saud University, KSA, through reference no. KSU-SE-19-51.

### 2.2. Animal Grouping and Experimental Design

44 animals were randomly divided into five groups (*n* = 8/9) comprising TBI animals treated with vehicle only (Group I), TBI animals treated with ketamine only (Group II), TBI animals treated to a combination of ketamine and perampanel treatment (Group III), sham animals were given vehicle only (Group IV), and sham animals treated with ketamine and perampanel (Group V).

On the 1^st^ day, animals were brought to the experimental room and acclimatized to this new environment for a few hours. Later, they were induced with brain injury by the weight drop method followed by group-wise-specific treatments after 30 minutes of the injury.

Animals of the vehicle-treated group were treated with normal saline only while ketamine (40 mg/kg, i.p.) and perampanel (4 mg/kg, p.o.) were given to the aforementioned groups. The doses of drugs and routes of administration were decided on the basis of previously reported studies related to ketamine [32, 33] and perampanel [34, 35].

**Figure 1:**
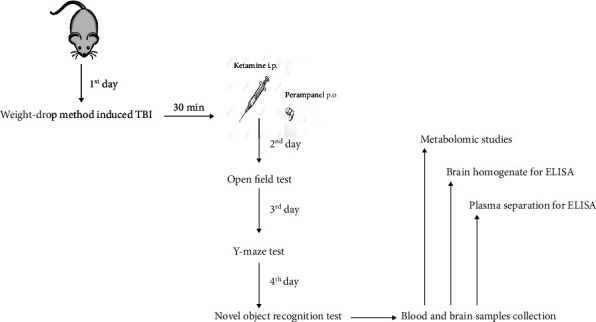
Experimental design depicting day-wise behavioral studies with subsequent in vitro analysis.

On the 2^nd^ day, after 24 h of TBI induction and subsequent treatments, the general locomotor activity was examined through the open field test. Afterward, the Y-maze and novel object recognition tests were performed on the 3^rd^ and 4^th^ day, respectively, to assess their learning and memory. Immediately after the completion of the behavioral test on the 4^th^ day, blood and brain samples were collected from randomly chosen animals. The blood samples were utilized for plasma estimation of NF-*κ*B and iNOS to check the impact of test drugs on systemic inflammation, while the brain homogenates were prepared to investigate the role of ketamine and perampanel on local inflammatory response after TBI (Figure 1).

### 2.3. Materials

Perampanel (Fycompa, 8 mg) and ketamine (Tekam, 10 mg/ml) were purchased from Eisai Co., Ltd. and Hikma Pharmaceuticals, respectively. Methoxyamine hydrochloride, pyridine, N,O-Bis(trimethylsilyl)trifluoroacetamide (BSTFA), and chlorotrimethylsilane (TMCS) were purchased from Sigma-Aldrich, St. Louis, MO, USA. Hexane and methanol were purchased from BDH VWR International Ltd. Poole, BH 15 1TD, England. Deionized water was obtained with Milli-RO and Milli-Q Plus instrumentation from Millipore, Billerica, MA, USA.

### 2.4. Induction of Brain Injury

Inducing brain trauma by the weight-drop method is well known for imitating human closed-head injury [36]. Thus, we employed this model of TBI in which severity of trauma can be controlled by adjusting the mass of weight and height from which it falls. The model utilized a weight-drop device that causes focal blunt trauma to one side of the unprotected skull. This results in damage to BBB and initiation of the neuroinflammatory cascade which collectively yields neurological deficit and behavioral impairment.

Initially, mice were exposed to isoflurane vapors (2-4 minutes) for anesthesia. The head of the animal was positioned on a platform under the device to direct the trauma from the left anterior frontal area at the same distance between the eye and the ear. A metallic mass of 30 g was guided through a hollow tube to fall freely on the targeted point from a height of 80 cm [37].

### 2.5. Behavioral Studies

#### 2.5.1. Open Field Test

The open field test is widely employed to evaluate the general exploratory activity in mice as they instinctively try to explore the open areas [38]. The test was conducted in a square-shaped (16^″^ × 16^″^) glass apparatus equipped with infrared sensors and perimeter. Mice were placed in this chamber and were allowed to acclimatize for 20 min to this environment. Afterward, their locomotion was recorded for 10 minutes to evaluate the total distance traveled and their preference for open areas of apparatus was evaluated to estimate the TBI-induced anxiety behavior in mice.

### 2.6. Behavioral Tests for Memory and Learning

#### 2.6.1. Y-Maze Test

In the Y-maze test, the animal's cognitive capacity is evaluated by monitoring its willingness to visit a new arm of maze and remembering the previously visited arm [39]. The test apparatus comprises three closed arms (35 cm × 5 cm × 10 cm) designed closely at 120°. The mice were placed separately in the center of the maze facing one of three arms (A, B, and C) keeping the apparatus cleaned with 70% alcohol after each animal's trial. The individual mouse was allowed to explore the maze for 5 minutes, and the sequence of the arm visits (ABC, BAC, CAB, etc.) was recorded to calculate the % spontaneous alternation behavior. An increased %SAP is an indication of better cognition and memory in rodents.

% spontaneous alternation was calculated by the following formula [40]:

(1) $$\begin{array}{c} \%\text{Spontaneous alternation}\left(\%\text{SAP}\right)=\left[\frac{\text{Number of alternations}}{\text{Total arm entries}-2}\right]\times 100. \end{array}$$

#### 2.6.2. Novel Object Recognition Test

This test is based on characteristics of the rodent to explore new environments and is extensively acknowledged to estimate their cognitive function. The animal with efficient memory recognizes the previously explored familiar object and expresses a spontaneous tendency to discover the new one. The test was conducted in a transparent acrylic glass box (16^″^ × 16^″^) in which animals were tested individually. Initially, each mouse was given 5 minutes to get familiar with the environment. After that, in the training phase, two geometrically identical objects were placed in the test box and the mouse was allowed to discover them for 10 min by touching and smelling them. Later, one of these two objects was replaced by a novel object of a different shape in test trial. The time spent with novel and familiar objects was recorded for 5 minutes to calculate the discrimination index:

(2) $$\begin{array}{c} \text{Discrimination index}=\left[\frac{\text{Time with novel object}-\text{Time with familiar object}}{\text{Time with novel object}+\text{Time with familiar object}}\right]. \end{array}$$

### 2.7. Blood Collection for Plasma Analysis

Soon after completion of the novel object recognition test, the blood of randomly taken animal (*n* = 3 per group) was collected in clean glass tubes through cardiac puncture. The blood samples were centrifuged at 3,000 rpm (800 *g*) for 10 min to separate the plasma to be analyzed which was stored at -80°C till further analysis [41].

Plasma levels of iNOS and NF-*κ*B were assessed through the commercially available ELISA kits and developed according to the manufacturer's protocols (iNOS ELISA Kit Cat. No. R6663, TSZELISA, Biotang Lab Supplier, USA; NF-*κ*B ELISA Kit Cat. No. 85-86081-11, Thermo Fisher Scientific, USA) for assessment of TBI and test treatment on their systemic expression.

### 2.8. Preparation of Brain Homogenate

After anesthetization with isoflurane, the brains of randomly selected animals (*n* = 3 per group) were dissected out and stored at 8°C in normal saline. 0.5 g of the brain was homogenized in 5 ml of phosphate buffer (pH 7.4) through centrifugation at 12,000 *g* for 15-20 minutes maintaining the low temperature of the mixture [42]. The resulting homogenized solution was evaluated through ELISA for any fluctuation in brain levels of inflammatory modulators, i.e., NF-*κ*B and iNOS, after traumatic brain injury and the impact of test drugs on these TBI-induced consequences.

### 2.9. Metabolomic Analysis

Metabolomic analysis was done with slight modifications in the method described [43]. The plasma samples were thawed at room temperature and vortex for 2 min. 100 *μ*l of this sample was put in the Eppendorf tube and 300 *μ*l of methanol and 100 *μ*l of Milli-Q water were added. Vortex again for 2 min and centrifuge for 10,000 rpm for 10 min at 4°C. From the supernatant solution, pipette out 200 *μ*l of sample and transfer it to a GC-MS vial. Purge the solution with nitrogen air to completely dry the vial. After this process, methoxylation was carried out at room temperature by adding 100 *μ*l of methoxyamine hydrochloride in pyridine solution (15 mg ml^−1^). The mixture was vortexed for 10 min and put the sample for half an hour at room temperature. In the second step, the methoxylated sample underwent a derivatization reaction by using 100 *μ*l of BSTFA/TMCS (99/1, *v*/*v*) and vortexed again for 10 min and kept for 2 hours at 50°C to complete the derivatizing reaction. 1 *μ*l of the derivatized sample was injected into the system with the split mode (split ratio 1 : 20).

A PerkinElmer model Clarus 600 T combined with a single quadrupole mass spectrometer was used for GC-MS analysis. The chromatographic column was an Elite 5MS column (30 m × 0.25 mm × 0.25 *μ*m film thickness), with high-purity helium as the gas carrier, at a flow rate of 1 ml/min. The injector temperature was 280°C and it was equipped with a splitless injector at 20 : 1. The temperature was set initially to 40°C (held for 2 min), was increased to 150°C at 10°C min^−1^ (held for 2 min), and then increased further to 300°C at 10°C min^−1^ (held for 2 min). The MS ion source temperature was 220°C, and inlet line temperature was at 240°C. The scan range was set at 40 to 600 mass ranges at 70 eV electron energy and the solvent delay of 7 min. Finally, unknown compounds were identified by comparing the spectra with that of the NIST 2005 (National Institute of Standard and Technology Library) and Wiley 2006 Library. The total time required for analyzing a single sample was 32 minutes.

### 2.10. Statistical Analysis

Data were analyzed through one-way ANOVA using GraphPad Prism 9 Software Inc. (La Jolla, CA, USA) followed by Sidek's or Dunnett's post hoc test. All arithmetic values were expressed as the mean ± SD considering *P* < 0.05 as statistically significant. Metabolomic data were analyzed via two-way ANOVA data that were presented as the mean ± SEM.

## 3. Results

### 3.1. Behavioral Analysis

#### 3.1.1. Effect of Administration of Ketamine Alone and in Combination with Perampanel on General Exploration and Anxiety after Traumatic Brain Injury

**Figure 2:**
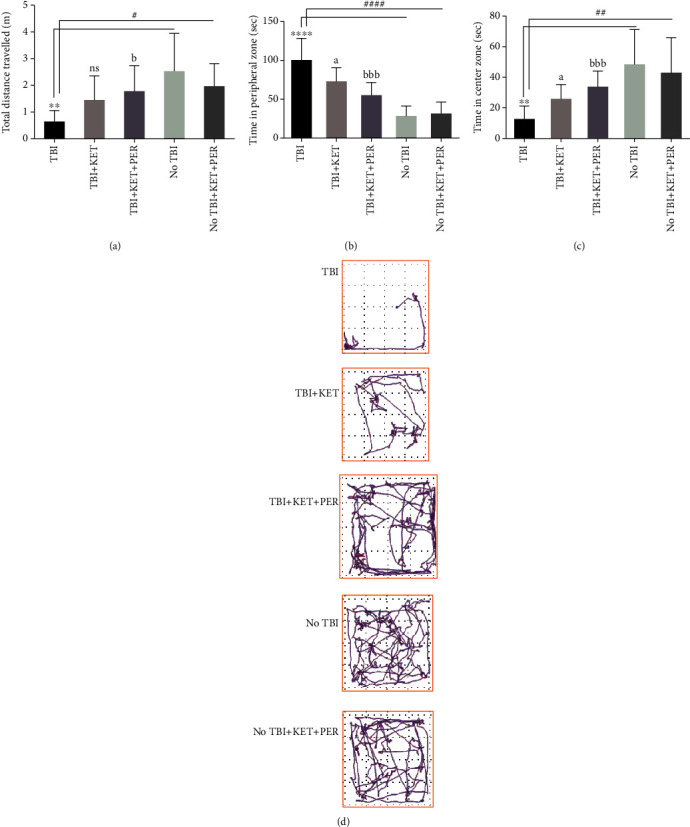
Comparison of total distance traveled (a), time spent in peripheral zone (b), time spent in central zone (c), and tracings of general exploratory behavior (d) among different groups in the open field test. ^∗∗^*P* < 0.01 and ^∗∗∗∗^*P* < 0.0001 show comparisons b/w TBI mice with healthy ones receiving vehicle only. ^#^*P* < 0.05, ^##^*P* < 0.01, and ^####^*P* < 0.0001 show comparisons b/w TBI mice with healthy ones exposed to ketamine-perampanel combination. ^a^*P* < 0.05 shows comparison b/w TBI mice with the TBI mice treated with ketamine only. ^b^*P* < 0.05 and ^bbbb^*P* < 0.0001 show comparisons b/w TBI mice with TBI mice treated with ketamine-perampanel combination. All data are expressed as the mean ± SD (*n* = 8/9 animals per group).

Animals with traumatic brain injury demonstrated lesser exploration of test apparatus than healthy mice (*P* < 0.01) and to those healthy mice treated with KET+PER (*P* < 0.05) revealing the brain trauma-induced motionlessness in animals (Figure 2(d)). However, the administration of KET+PER reversed these outcomes as total distance traveled was notably increased (*P* < 0.05) than TBI mice. However, the administration of ketamine did not yield significant outcomes on exploration (Figure 2(a)).

However, TBI caused animals to act anxiously as they displayed partialities towards marginal areas of the field and spent more time their (*P* < 0.0001) than the central zone (*P* < 0.01). Interestingly, the anxiety was reduced through the administration of ketamine as animals spent more time in the central (*P* < 0.05) and less time in peripheral (*P* < 0.05) zones of test apparatus. The outcomes were even more pronounced on the administration of KET+PER as the animals were unafraid and preferred center zone (*P* < 0.001) over the peripheral zone as compared to TBI mice (Figures 2(b) and 2(c)).

#### 3.1.2. Effect of Administration of Ketamine Alone and in Combination with Perampanel on TBI-Induced Cognitive Deficit in the Y-Maze Test

**Figure 3:**
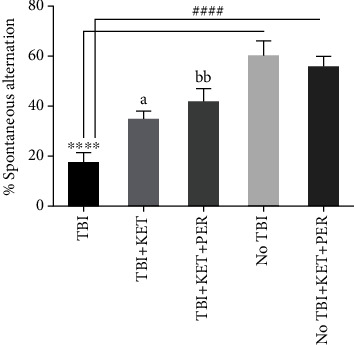
Comparison of % spontaneous alternation behavior among different groups in the Y-maze test. ^∗∗∗∗^*P* < 0.0001 shows comparisons b/w TBI mice with healthy ones receiving vehicle only. ^####^*P* < 0.0001 shows comparisons b/w TBI mice with healthy ones exposed to ketamine-perampanel combination, ^a^*P* < 0.05 shows comparison b/w TBI mice with the TBI mice treated with ketamine only. ^bb^*P* < 0.01 shows comparisons b/w TBI mice with TBI mice treated with ketamine-perampanel combination. All data are expressed as the mean ± SD (*n* = 8/9 animals per group).

The Y-maze test was employed to evaluate the influence of investigational drugs on cognitive decline in TBI mice. The mice were not capable enough of recalling the previously visited arm of maze due to neuronal damage resulting from physical injury to the brain. The experimental outcomes demonstrated memory scarcity as evident from reduced alternation behavior (*P* < 0.0001) than both groups of healthy mice. This cognitive deficit was improved when animals were treated with ketamine (*P* < 0.05) and ketamine-perampanel combination (*P* < 0.01) showing that the treatments led to improved spatial remembrance as they significantly reversed the intellectual impairment in mice resulting from TBI (Figure 3).

#### 3.1.3. Effect of Administration of Ketamine Alone and in Combination with Perampanel on TBI-Induced Cognitive Deficit in the Novel Object Recognition Test

**Figure 4:**
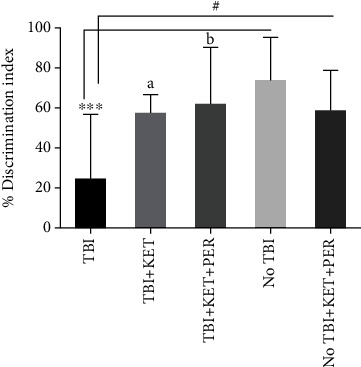
Comparison of % discrimination index among different groups in the novel object recognition test. ^∗∗∗^*P* < 0.001 shows comparisons b/w TBI mice with healthy ones receiving vehicle only. ^#^*P* < 0.05 shows comparisons b/w TBI mice with healthy ones exposed to ketamine-perampanel. ^a^*P* < 0.05 shows comparison b/w TBI mice with the TBI mice treated with ketamine only. ^b^*P* < 0.05 shows comparisons b/w TBI mice with TBI mice treated with ketamine-perampanel combination. All data are expressed as the mean ± SD (*n* = 8/9 animals per group).

This test was used to evaluate the memory of mice exposed to brain trauma and the impact of investigational treatments on their behavior. The mice with TBI revealed poor intellect as they could not retain the memory of the familiar object and exhibited lower discrimination index as compared to vehicle-treated (*P* < 0.001) and ketamine-perampanel-treated (*P* < 0.05) healthy mice. This cognitive deficit was significantly improved on exposure to ketamine (*P* < 0.05) and ketamine-perampanel combination (*P* < 0.05) as animals retained the familiarity of previously explored objects and preferred the novel one (Figure 4).

### 3.2. Effect of Administration of Ketamine Alone and in Combination with Perampanel on Plasma Levels of NF-*κ*B and iNOS after Traumatic Brain Injury

**Figure 5:**
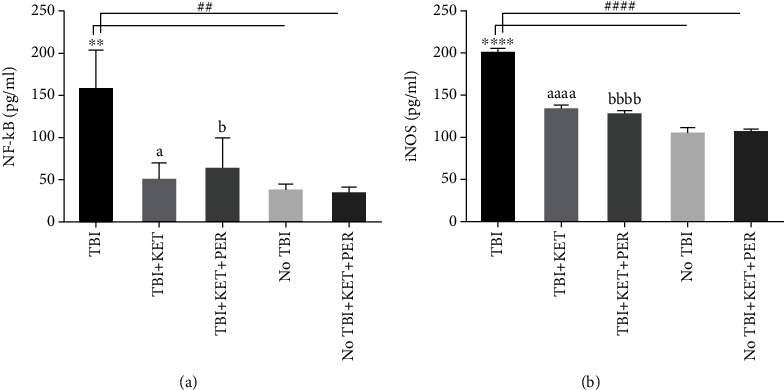
Comparison of plasma levels of NF-*κ*B (a) and iNOS (b) among differently treated groups through ELISA. ^∗∗^*P* < 0.01 and ^∗∗∗∗^*P* < 0.0001 show comparisons b/w TBI mice with healthy ones receiving vehicle only. ^##^*P* < 0.01 and ^####^*P* < 0.0001 show comparisons b/w TBI mice with healthy ones exposed to ketamine-perampanel. ^a^*P* < 0.05 and ^aaaa^*P* < 0.0001 show comparison b/w TBI mice with the TBI mice treated with ketamine only. ^b^*P* < 0.05 and ^bbbb^*P* < 0.0001 show comparisons b/w TBI mice with TBI mice treated with ketamine-perampanel combination. All data are expressed as the mean ± SD (*n* = 3 animals per group).

Plasma levels of NF-*κ*B were estimated to check the impact of test drugs on this proinflammatory mediator as it acts as a cofactor in transcription and signaling pathways in systemic inflammation after some injury. In the current study, ELISA exposed the elevated levels of NF-*κ*B after TBI as compared to healthy animals (*P* < 0.01) and to those healthy mice treated with ketamine-perampanel combination (*P* < 0.01). These TBI-elevated plasma concentrations of NF-*κ*B were reduced significantly in mice treated with ketamine (*P* < 0.05) and with ketamine-perampanel combination (*P* < 0.05) (Figure 5(a)). Similarly, plasma levels of iNOS were also increased in TBI mice (*P* < 0.0001) as brain injury is also known to increase the inflammatory NO in distant organs as well as to yield further complications after initial injury. However, the administration of ketamine reversed (*P* < 0.001) these detrimental outcomes of TBI (Figure 5(b)) and the parallel result was seen in animals treated with ketamine-perampanel combination (*P* < 0.0001).

### 3.3. Effect of Administration of Ketamine Alone and in Combination with Perampanel on Concentration of NF-*κ*B and iNOS in the Brain after Traumatic Brain Injury

**Figure 6:**
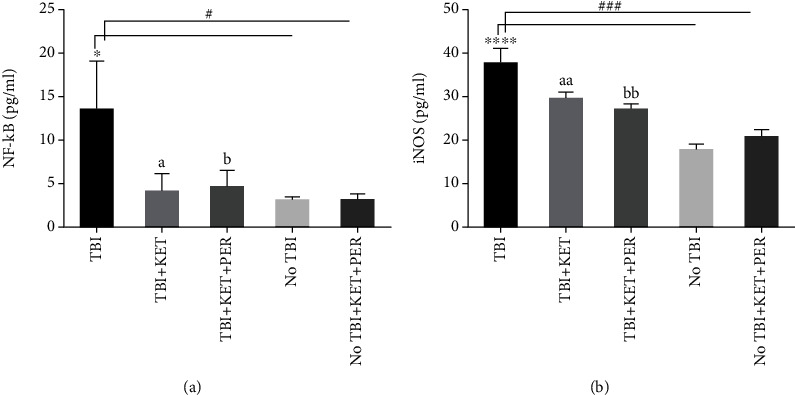
Evaluation of concentration of NF-*κ*B (a) and iNOS (b) in the brains of differently treated groups after traumatic brain injury. ^∗^*P* < 0.05 and ^∗∗∗∗^*P* < 0.0001 show comparisons b/w TBI mice with healthy ones receiving vehicle only. ^#^*P* < 0.05 and ^###^*P* < 0.001 show comparisons b/w TBI mice with healthy ones exposed to ketamine-perampanel. ^a^*P* < 0.05 and ^aa^*P* < 0.01 show comparison b/w TBI mice with the TBI mice treated with ketamine only. ^b^*P* < 0.05 and ^bb^*P* < 0.01 show comparisons b/w TBI mice with TBI mice treated with ketamine-perampanel combination. All data are expressed as the mean ± SD (*n* = 3 animals per group).

ELISA revealed the raised levels of NF-*κ*B in the brain after a traumatic injury as compared to healthy brains (*P* < 0.05), and these detrimental outcomes were resolved after treatment with ketamine alone (*P* < 0.05) and in combination with perampanel (*P* < 0.05) (Figure 6(a)).

Likewise, iNOS expression was also enhanced in the brains of the TBI group as compared to the brains dissected from healthy mice receiving no treatment (*P* < 0.0001) and receiving ketamine-perampanel (*P* < 0.001). This elevated expression of iNOS was normalized when TBI mice were treated with ketamine (*P* < 0.01) and ketamine-perampanel combination (*P* < 0.01) (Figure 6(b)).

### 3.4. Outcomes of Administration Combination Therapy on with Perampanel on Metabolomics

**Figure 7:**
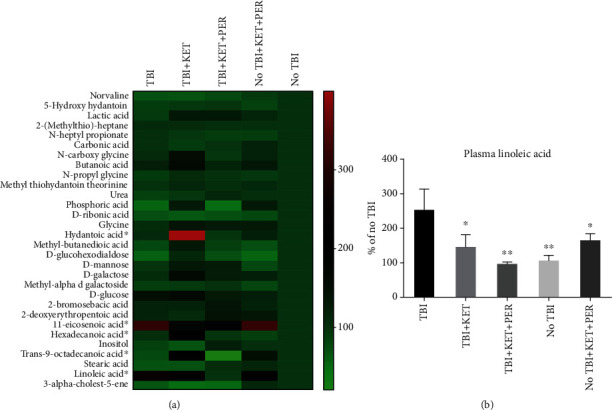
Plasma metabolomic analysis. A heat map shows thirty identified plasma metabolites (a) and differences in plasma concentrations of linoleic acid (b). The statistically significant changed metabolites were labeled bold on the heat map (a). All data are expressed as the mean ± SEM (*n* = 3‐4). Outcomes of all groups were compared with the No TBI group which is assumed 100%, and comparative statistical significance ^∗^*P* < 0.05 was compared with the TBI group. Two-way ANOVA analysis of normalized values (as percentage of the No TBI group). ^∗^*P* < 0.05 and ^∗∗^*P* < 0.01 (Supplementary Raw Data).

The plasma metabolomic profile was assessed to further explore the effect of our treatment on TBI. Thirty metabolites were found (Figure 7(a)), which are linked to various pathways including galactose metabolism, biosynthesis of unsaturated fatty acids, amino sugar, and nucleotide sugar metabolism. To assess the differences between our groups, two-way ANOVA was performed to statistically identify the differences between our groups. Among these thirty, five metabolites were significantly changed among groups including hydantoic acid, 11-eicosenoic acid, hexadecanoic acid, trans-9-octadecanoic acid, and linoleic acid revealing uniqueness in its metabolomic profile of each group. However, linoleic acid was the one that got significantly increased after TBI and this outcome was prominently neutralized by combination therapy (Figure 7(b)) suggesting its role in the findings of our study.

## 4. Discussion

Traumatic brain injury affects millions of people every year through continual disabilities and mortalities in almost all age groups from all over the world [44]. Epidemiological estimations predict that TBI-resulted ceaseless debilitation rate will leave the other ailment-imparted incapacities behind in the coming decade [45]. Unfortunately, the current advancement in trauma research through the collaboration of multidisciplinary professionals is still insufficient to handle this challenge and TBI continues to be a major burden on global health and patient's income.

After any physical damage to the brain, the gradual pathophysiological fluctuations take place continuously resulting in reduced blood flow and oxygenation of brain tissues leading to BBB damage and edema [46]. The second phase of damage causes more worsening of neurological function and may start after minutes or weeks to primary injury. Various pathways that play a role in this biphasic damage are the excessive release of glutamate, generation of free radicals due to mitochondrial dysfunction, and neuroinflammation due to local and systemic immunoactivation [47].

The amino acid glutamate is released at up to half of synapses in the brain and is known as the main excitatory neurotransmitter in the CNS which can act as excitotoxin as of NMDA and AMPA receptors can result into neuronal damage. Currently, glutamatergic storming is accepted to play a critical role in grading the severity of TBI [23]. Ketamine attenuates this excitotoxicity by exhibiting the noncompetitively antagonizing NMDA receptor. Furthermore, it also improves cerebral vascular flow at subanesthetic doses and addresses the root cause of excitotoxicity [48]. Ketamine also causes inhibition of neurotoxic NR2B-comprising NMDA receptors and this plays a friendly role in neuronal regeneration [49]. On the other side, perampanel rescues the tissue from damage by antagonizing the AMPA-induced increases in intracellular Ca^2+^ which plays a role in glutamatergic storming [50]. It also plays anti-inflammatory action by suppressing the expression of TNF-*α* and IL-1*β* (proinflammatory cytokines) and enhancing the expression IL-10 and TGF-*β*1 (anti-inflammatory cytokines) [26]. These might be the mechanisms through which the ketamine and perampanel reversed the deteriorating effects of TBI.

The brain is highly susceptible to oxidative stress due to its high content of polyunsaturated lipids. As physical trauma results in impaired circulation and oxygenation of the brain, there is an increased generation of ROS including nitric oxide and superoxide [51]. Peroxynitrite is another strong oxidant generated due to the reaction of nitric acid with superoxide and causes further impairment in cerebral vascular function after TBI [52]. This process depletes the natural antioxidants and peroxidation of lipid membrane and disruption of mitochondrial electron transport system results in necrosis and apoptosis. The Nrf2 pathway has been known as a cellular defense mechanism against oxidative stress that works by upregulating the phase II enzymes [53]. Ketamine administration after TBI exerts a neuroprotective role by combating the oxidative stress through a significant increase in Nrf2 expression [33]. Additionally, a study has reported the beneficial effect of perampanel through inhibition of lipid peroxidation and preservation of endogenous antioxidant reservoirs that work to inhibit TBI-induced oxidative stress [26].

Local tissue injury can provoke the inflammation through iNOS expression in cells of vascular smooth muscles [54] as well as in brain immune cells and endothelial cells at BBB [55]. The macrophages expressing iNOS can play a role in the activation of inflammatory events leading to dysfunction of the blood-brain barrier, and these consequences contribute to the second phase of TBI [56]. The regulation of iNOS takes place at the transcriptional level, and the promoter region of iNOS gene contains various binding sites for transcriptional factors including NF-*κ*B [57]. In our study, treatment of TBI mice with ketamine and ketamine-perampanel combination demonstrated a significant reduction in local and systemic expression of NF-*κ*B and iNOS. This might be due to the antioxidant potential of ketamine [33] and perampanel [26] as oxidative stress plays a crucial role in the activation of NF-*κ*B in different cell types [57].

Though a range of behavioral changes is seen as post-TBI difficulties, impaired cognition and deteriorated learning are commonest consequences [58]. In the present study, the TBI mice exhibited impaired memory by demonstrating reduced alternation behavior in the Y-maze and poor object identification in the novel object recognition test. However, these outcomes were improved by ketamine and ketamine-perampanel combination. Similarly, the animal testing through the open field test revealed that TBI mice stayed stationary and anxious with less exploration of field and avoidance of open areas of apparatus. Treatment with ketamine-perampanel combination improved the TBI-deteriorated behavior, and animals showed improved learning and fearlessness.

Various studies have reported the association of increased ROS and anxiety [59, 60]. Oxidative stress also results in cognitive decline as seen in the normal aging process too [61]. Ketamine and perampanel drugs impart an inhibitory effect on NF-*κ*B leading to reduced iNOS expression [62]. These antioxidant influences of test drugs can be the mechanism through which they improved outcomes in the behavioral tests. Besides this, ketamine is known to alter hippocampal cell proliferation as NMDARs play a role in modulating neurogenesis after TBI [31]. Additionally, perampanel is also reported to exert the cognition-boosting effect by reduced neuronal apoptosis [26].

Linoleic acid is one of the major polyunsaturated fatty acids (PUFA) in mammals which acts as a substrate for lipoxygenase [63], and the lipid mediators produced are reported to implicate BBB damage, activation of inflammatory reactions, and dysregulated cerebral flow [64]. It can be nonenzymatically autoxidized to its hydroperoxides that work as a source of massive oxidation damaging reactions in the biological systems [65]. GC/MS-based nontargeted metabolomics conducted on separated plasma revealed the elevated level of linoleic acid after acute TBI as previously reported as well [43]. Linoleic acid is the part of membrane phospholipids [66] and its elevated levels were reduced by ketamine-perampanel combination illustrating their role to reduce the damage to the cellular membrane either through combating the excitotoxicity or exerting an antioxidant effect.

## 5. Conclusion

In the present study, the administration of a subanesthetic dose of ketamine alone and in combination with perampanel showed retrieval from a post-TBI behavioral deficit in C57BL/6J mice. The TBI-resulted overexpression of local and systemic inflammatory modulators, NF-*κ*B and iNOS, was positively regulated by test drug combination as well as ketamine alone. Furthermore, metabolomic studies also supported the neuroprotective impact of therapy as the reversal of metabolic changes was observed. These beneficial outcomes might go through antagonizing the glutamatergic storming or through suppressing the local as well as systemic expression of NF-*κ*B and iNOS. The study unveiled the rescuing effect of the NMDA receptor antagonist, ketamine, on TBI, and the outcomes were even much improved by combining it with the AMPA receptor antagonist, perampanel. This newly investigated polypharmacy could be used as a therapeutic approach in the management of traumatic brain injury.

## Data Availability

The raw data used to support the findings of this study are available from the corresponding author upon request.
